# Effects of olive oil and its minor phenolic constituents on obesity-induced cardiac metabolic changes

**DOI:** 10.1186/1475-2891-9-46

**Published:** 2010-10-19

**Authors:** Geovana MX Ebaid, Fábio RF Seiva, Katiucha KHR Rocha, Gisele A Souza, Ethel LB Novelli

**Affiliations:** 1 Department of Chemistry and Biochemistry, Institute of Biological Sciences, São Paulo State University, UNESP, 18618-000, Botucatu, São Paulo, Brazil; 2 Post Graduation Course Department of Clinical and Cardiology, School of Medicine, São Paulo State University, UNESP, Botucatu, São Paulo, Brazil

## Abstract

**Background:**

Olive oil and its minor constituents have been recommended as important dietary therapeutic interventions in preventive medicine. However, a question remains to be addressed: what are the effects of olive oil and its phenolic compounds on obesity-induced cardiac metabolic changes?

**Methods:**

Male Wistar rats were divided into two groups (*n *= 24/group): (C) receiving standard-chow; (Ob) receiving hypercaloric-chow. After 21 days C and Ob groups were divided into four subgroups (*n *= 6/group):(C) standard-chow and saline; (C-Olive)standard-chow and olive-oil (3.0 g/kg.day); (C-Oleuropein)standard-chow and oleuropein (0.023 mg/kg/day); (C-Cafeic) standard-chow and cafeic-acid (2.66 mg/kg/day); (Ob)receiving hypercaloric-chow and saline;(Ob-Olive) hypercaloric-chow and olive-oil;(Ob-Oleuropein) hypercaloric-chow and oleuropein;(Ob-Cafeic) hypercaloric-chow and cafeic-acid. Treatments were given twice a week during 21 days.

**Results:**

After 42 days, obesity was evidenced in Ob rats from enhanced body-weight, surface-area, and body-mass-index. Energy-expenditure, oxygen consumption(VO_2_) and fat-oxidation were lower in Ob-group than in C. Despite no morphometric changes, Ob-Olive, Ob-Oleuropein and Ob-Cafeic groups had higher VO_2_, fat-oxidation, myocardial beta-hydroxyacyl coenzyme-A dehydrogenase and lower respiratory-quotient than Ob. Citrate-synthase was highest in Ob-Olive group. Myocardial lipid-hydroperoxide(LH) and antioxidant enzymes were unaffected by olive-oil and its compounds in obesity condition, whereas LH was lower and total-antioxidant-substances were higher in C-Olive and C-Oleuropein than in C.

**Conclusions:**

The present study demonstrated for the first time that olive-oil, oleuropein and cafeic-acid enhanced fat-oxidation and optimized cardiac energy metabolism in obesity conditions. Olive oil and its phenolic compounds improved myocardial oxidative stress in standard-fed conditions.

## Background

Olive oil consumption was claimed as the basic food supply responsible for the low incidence of cardiovascular diseases in some societies [1]. Recent research has suggested that olive oil minor constituents might have more effects on health than once believed [1-5].

On this concern, a group of olive oil phenolic compounds, such as oleuropein and cafeic acid have attracted considerable attention because of their anti-diabetic [6,7], anti-atherosclerotic [8] and anti-inflammatory [9] properties. Olive oil minor components have been also associated with the antioxidant activity of olive oil [9,10], but little information is available on cardiac oxidative stress and energy metabolism in obesity conditions.

Cardiac muscle utilizes a variety of substrates to produce energy, and the heart can shift from one substrate to another depending on food intake and pathophysiological state [11]. There is a growing awareness that during energy metabolism, the mitochondrial respiratory chain represents a major intracellular source of reactive oxygen species (ROS). Alterations in food constituents or substrate for energy generation, as found in hypercaloric diet [12] may result in higher ROS, thus inducing oxidative stress, an imbalance between oxidants and antioxidants systems in favor of the former [13,14]. Recent research in our laboratory demonstrated that enhanced energy intake reduced the mitochondrial membrane fluidity, increasing ROS generation [12,15].

Furthermore, it has been shown that blocking mitochondrial oxidative phosphorylation system increases lipid accumulation in adipocytes, and that in obesity there is decreased oxygen consumption, indicating impairment in mitochondrial activity [16]. Therefore, weight gain can be viewed not only as the consequence of an initial positive energy balance, but also as the mechanism by which energy balance is eventually stored. Whether obesity-related cardiac effects are due to an increase in caloric intake, a decrease in metabolic rate, or a combination of both factors is still unclear.

An excess of energy intake and/or a decrease in energy expenditure may reflect important metabolic dysfunction, for instance exacerbated fat storage and/or a deficit in oxidative metabolism [17]. However, it was not studied whether olive oil intake can alter calorimetric parameters and can improve energy metabolism in cardiac tissue of obese rats, which certainly would bring new insights on obesity-related cardiac metabolic changes control.

Thus, the aim of this study was to investigate the effects of olive oil and its minor phenolic constituents, oleuropein and cafeic acid, on calorimetric parameters, myocardial oxidative stress and energy metabolism in cardiac tissue of control and obese rats. Animal models are useful for increasing the understanding of the effects of diet and the development of obesity related abnormalities, particularly on cardiac tissue [15,18]. Therefore, we used a rat model to study the effects of olive oil and its phenolic compounds on an unresolved issue in the field of obesity and health that is the control of obesity-related cardiac metabolic alterations.

## Methods

### Obesity induction and experimental procedure

All experiments and procedures were performed in accordance with the Guide for the Care and Use of Laboratory Animals published by the US National Institute of Health and were approved by the Ethics Committee of the Botucatu School of Medicine, UNESP, Botucatu, SP, Brazil. Forty eight male Wistar rats, 75 days of age, were individually housed in polypropylene cages in an environmentally controlled clean-air room with a temperature of 22 ± 3°C, a 12 h light-dark cycle and a relative humidity of 60 ± 5%. Taking into account the hormonal influences in female, we used only male rats.

Initially the animals were randomly divided into two groups (*n *= 24/group). The (C) group received water *ad libitum *and standard rodent chow (3074 SIF, Purina Ltda., Campinas, SP, Brazil), containing (by weight) 19.80% protein, 39.25% carbohydrate, 4.41% fat, 13.25% fibre, and 2.76 kcal/g of metabolizable energy. The (Ob) group received a hypercaloric chow containing 15.25% protein, 43.34% carbohydrate, 11.86% fat, 10.20% fibre (by weight) and 3.41 kcal/g of metabolizable energy.

The hypercaloric chow was made mixing 131.01 g sucrose, 84.77 g of soy oil, 12.33 g of cholesterol and 1.23 g cholic acid with 1000 g of a previously triturated standard chow. The dietary ingredients were homogenized in 60°C warm distilled water and the homogenate was used to prepare the pellets. Therefore, both control and hypercaloric diets were given as pellets, and there was no spillage. The proportions of dietary protein, carbohydrate and fat were determined by direct analysis [19]. Food and drinking solutions consumption were measured daily at the same time (9:00-10:00 h). The body weights were determined once a week.

In order to more appropriately study the effects of olive oil and its minor constituents on obesity, and standard fed conditions, after 21 days of dietary treatments, both C and Ob groups were then randomly divided into four subgroups (n = 6/group): (C) remained to eat standard chow, receiving saline (NaCl 0,9%); (C-Olive) remained to eat standard chow, receiving olive oil supplementation (3.0 g/kg/day) [20,21]; (C-Oleuropein) remained to eat standard chow, receiving oleuropein (0.023 mg/kg/day) [22]; (C-Cafeic) remained to eat standard chow, receiving cafeic acid (2.66 mg/kg/day) [23]; (Ob) remained to eat hypercaloric chow, receiving saline (NaCl 0,9%); (Ob-Olive) remained to eat hypercaloric chow, receiving olive oil (3.0 g/kg/day); (Ob-Oleuropein) remained to eat hypercaloric chow, receiving oleuropein (0.023 mg/kg/day); (Ob-Cafeic) remained to eat hypercaloric chow, receiving cafeic acid (2.66 mg/kg/day). Treatments were given twice a week by gavage.

Extra-virgin olive oil was chosen for its high content of phenolic compounds and was purchased from the local market (Botucatu city, São Paulo State, Brazil). The olive oil administration was the adopted dose according to human olive oil consumption, and considered that the average consumption of olive oil in humans is 4% of the diet, twice a week [24]. This value corresponded to 1 g olive oil per 25 g of consumed chow, or 3 g/kg/day olive oil for rats of C-Olive and Ob-Olive groups [21]. The olive oil was analysed for oleuropein [25] and cafeic acid [26]. The oleuropein and cafeic acid doses took into account that the amount of these compounds in the olive oil utilized was 7.78 mg/l oleuropein and 887.5 mg/l cafeic acid. Since the amount of 3 g/kg/day olive oil [20,21] was given to the rats of control group, we administered 0.023 mg/kg/day oleuropein and 2.66 mg/kg/day cafeic acid, twice a week. Oleuropein and cafeic acid were purchased from Sigma (St. Louis, MO, USA). Food intake and caloric value of chows were used to obtain total energy intake.

### Indirect calorimetry

After 21 days of dietary supplementations (42 days of the experimental period), the rats were fasted overnight (12 to 14 h) and placed into metabolic chambers (airflow = 1.0 l/min) of a computer-controlled indirect calorimeter (CWE, Inc, St. Paul, USA) to determine the calorimetric parameters. Respiratory quotient (RQ) and energy expenditure, namely resting metabolic rate (RMR) were measured using a respiratory-based software program (software MMX, CWE, Inc., USA). Average oxygen consumption (VO_2_) and average carbon dioxide production (VCO_2_) were integrated over periods of 15 min. Carbohydrate and fat oxidation were calculated from the no protein oxygen consumption and the amount of oxygen consumed per gram of substrate oxidized. The energy balance over 24 h was determined as energy intake minus energy expenditure [27].

### Morphometric and biochemical determinations

After 42 days of experimental period, rats were fasted overnight (12 to 14 h). The animals were anaesthetized (0.1 ml ip of 1% sodium barbiturate) for measurement of body length (nose-to-anus, or nose-anal length) [19]. The body weight and body length were used to determine body mass index (BMI, body weight/body length^2^) and surface area (body weight^0.7^). After measuring the body length, the rats were sacrificed by decapitation. The heart was rapidly removed and weighed. The left ventricle samples of 200 mg were homogenized in 5 mL of a cold 0.1 M phosphate buffer, pH7.4. Tissue homogenates were prepared in a motor-driven Teflon-glass Potter-Elvehjem, tissue homogeniser (1 min, 100 × *g*). The homogenate was centrifuged at 10000 × *g*, for 15 min, and the supernatant was assayed for total protein [28], lipid hydroperoxide (LH) [29], antioxidant capacity, or total antioxidant substances (TAS) (test-kit Randox Laboratories, Crumlin, Co., Antrim, United Kingdom), glutathione peroxidase (GSH-Px, E.C.1.11.1.9) [30], catalase (E.C.1.11.1.6.) [31] and superoxide dismutase (SOD, E.C.1.15.1.1.) [32].

Several key metabolic enzymes were studied to reveal the possible alteration of myocardial function due obesity. The cardiac energy metabolism was assessed by lactate dehydrogenase (LDH; E.C.1.1.127.), beta-hydroxyacyl coenzyme-A dehydrogenase (OHADH; E.C.1.1.1.35.) and citrate synthase (CS; E.C.4.1.3.7.) determinations [33].

Enzyme activities were performed at 25°C using a micro-plate reader (μQuant-MQX 200 with Kcjunior software to computer system control, Bio-Tec instruments, Winooski, Vermont, USA). The spectrophotometric determinations were performed in a Pharmacia Biotech spectrophotometer with temperature-controlled cuvette chamber (UV/visible Ultrospec 5,000 with Swift II applications software to computer system control, 974213, Cambridge, England, UK).

### Statistical analysis

The results are presented as means ± standard deviations (S.D). Analysis of variance for two variables (Two Way-ANOVA) was used to examine the diet and supplementation treatment effects. Significant analysis of variance results were subjected to post hoc Tukey's test. Statistical significance was set at *p *< 0.05 (Systat Software, USA).

## Results

### General characteristics of rats, nutritional and calorimetric parameters

Hypercaloric diet significantly increased final body weight, BMI and surface area. Despite no changes in food consumption, the energy intake was higher in Ob than in C rats. Ob rats had depressed VO_2, _VO_2_/body weight, VO_2_/surface area, RMR, RMR/body weight and fat oxidation, as well enhanced RQ, RQ/surface area, carbohydrate oxidation and energy balance, comparing with C rats (Table 1). Olive oil, oleuropein and cafeic acid in control-fed and obese-rats had no effects on final body weight, surface area, BMI, food consumption and energy intake.

**Table 1:** General characteristics and calorimetric parameters of rats after 42 days of standard fed (C), and hypercaloric fed (Ob) diets

	Groups	
	
Parameters	C	Ob
Final body weight (g)	335.75 ± 54.32	397.66 ± 36.54^a^
Surface area (g^0.7^)	58.51 ± 6.60	65.96 ± 4.29^a^
BMI (g/cm^2^)	0.54 ± 0.03	0.65 ± 0.05^a^
Food consumption (g/day)	25.4 ± 4.6	25.3 ± 1.4
Energy intake (kcal/day)	70.07 ± 4.30	86.27 ± 4.09^a^
VO_2 _(ml/min)	3.80 ± 0.06	3.46 ± 0.11^a^
VCO_2 _(ml/min)	2.35 ± 0.001	2.49 ± 0.002^a^
RQ	0.63 ± 0.04	0.84 ± 0.03^a^
RMR (kcal/h)	1.07 ± 0.02	0.97 ± 0.01^a^
VO_2_/body weight (ml/h.kg)	11.58 ± 1.78	8.75 ± 0.53^a^
VO_2_/surface area (mL/h.g^0.7^)	3.94 ± 0.42	3.15 ± 0.12^a^
RQ/body weight	0.19 ± 0.03	0.21 ± 0.02
RQ/surface area	1.10 ± 0.02	1.28 ± 0.10^a^
RMR/body weight (kcal/h.kg)	3.25 ± 0.04	2.46 ± 0.23^a^
Fat oxidation (mg/min)	3.50 ± 0.39	1.38 ± 0.28^a^
Carbohydrate oxidation (mg/min)	-	1.19 ± 0.25^a^
Energy balance (kcal/day)	49.65 ± 5.51	62.93 ± 4.27ª

Values are given as mean ± SD.

C, control rats; Ob, obese rats; BMI, body mass index; VO_2 _oxygen consumption; VCO_2 _dioxide carbon production; RQ, respiratory quotient; RMR, resting metabolic rate.

^a^*p *< 0.05 vs. C group.

C-Olive and C-Cafeic rats had higher VO_2_, VCO_2_, RMR and fat oxidation than C. VO_2_/surface area, RQ and RQ/surface area were higher in C-Olive than in C. C-Oleuropein rats had lower VO_2_, RMR and fat oxidation, as well higher RQ and RQ/surface area than C-Olive. C-Cafeic rats had higher VO_2_, RMR and fat oxidation, as well lower RQ, RQ/body weight and RQ/surface area than C-Oleuropein (Table 2). Ob-Olive, Ob-oleuropein and Ob-Cafeic rats had lower RQ, RQ/body weight and RQ/surface area, as well higher fat oxidation than Ob. The VO_2 _and energy balance were higher, while RMR and RMR/body weight were lower in Ob-Cafeic than in Ob, Ob-Olive and Ob-Oleuropein rats (Table 3). The carbohydrate oxidation was not detected in C, C-Olive and C-Cafeic, as well in Ob-Olive, Ob-Oleuropein and Ob-Cafeic rats (Tables 2 and 3).

**Table 2:** Calorimetric parameters of standard-fed rats.

	Groups			
	
Calorimetric parameters	C	C-Olive	C-Oleuropein	C-Cafeic
VO_2 _(ml/min)	3.80 ± 0.06	4.77 ± 0.37^a^	3.44 ± 0.09^b^	4.45 ± 0.26^ac^
VCO_2 _(ml/min)	2.35 ± 0.001	2.36 ± 0.002^a^	2.37 ± 0.002^ab^	2.38 ± 0.002^abc^
RQ	0.63 ± 0.04	0.54 ± 0.04^a^	0.78 ± 0.03^b^	0.60 ± 0.03^c^
RMR (kcal/h)	1.07 ± 0.02	1.21 ± 0.09^a^	0.97 ± 0.04^ab^	1.18 ± 0.06^ac^
VO_2_/body weight (ml/h.kg)	11.58 ± 1.78	13.46 ± 1.45	10.13 ± 1.21^b^	12.02 ± 1.17
VO_2_/surface area (mL/h.g^0.7^)	3.94 ± 0.42	4.69 ± 0.38^a^	3.49 ± 0.31^a^	4.25 ± 0.33^b^
RQ/body weight	0.19 ± 0.03	0.15 ± 0.02^a^	0.23 ± 0.02^b^	0.16 ± 0.01^c^
RQ/surface area	1.10 ± 0.02	0.88 ± 0.08^a^	1.31 ± 0.1^ab^	0.95 ± 0.07^c^
RMR/body weight (kcal/h.kg)	3.25 ± 0.04	3.44 ± 0.55	2.85 ± 0.36	3.19 ± 0.30
Fat oxidation (mg/min)	3.50 ± 0.39	5.55 ± 0.49^a^	1.92 ± 0.35^ab^	4.55 ± 0.64^abc^
Carbohydrate oxidation (mg/min)	Not detected	Not detected	0.64 ± 0.33	Not detected
Energy Balance (kcal/day)	49.65 ± 5.51	50.90 ± 8.86	52.49 ± 7.37	54.66 ± 9.92

Values are given as mean ± SD.

C, control rats receiving saline; C-Olive, standard-fed rats receiving olive oil; C-Oleuropein, standard-fed rats receiving oleuropein; C-Cafeic, standard-fed rats receiving cafeic acid; VO_2 _oxygen consumption; VCO_2_, dioxide carbon production; RQ, respiratory quotient; RMR, resting metabolic rate.

^a^*p *< 0.05 vs. C group.

^b ^*p *< 0.05 vs. C-Olive group.

^c ^*p *< 0.05 vs. C-Oleuropein group.

**Table 3:** Calorimetric parameters of obese rats.

	Groups			
	
Calorimetric parameters	Ob	Ob-Olive	Ob-Oleuropein	Ob-Cafeic
VO_2 _(ml/min)	3.46 ± 0.11	3.60 ± 0.27	3.78 ± 0.23	4.00 ± 0.14^ab^
VCO_2 _(ml/min)	2.49 ± 0.002	2.47 ± 0.002^a^	2.47 ± 0.014^a^	2.48 ± 0.005^c^
RQ	0.84 ± 0.03	0.64 ± 0.03^a^	0.70 ± 0.11^a^	0.66 ± 0.03^a^
RMR (kcal/h)	0.97 ± 0.01	0.97 ± 0.04	1.05 ± 0.06	0.42 ± 0.07^abc^
VO_2_/body weight (ml/h.kg)	8.75 ± 0.53	9.39 ± 1.35	9.49 ± 0.88	10.12 ± 0.93
VO_2_/surface area (ml/h.g^0.7^)	3.15 ± 0.12	3.35 ± 0.42	3.43 ± 0.29	3.64 ± 0.27
RQ/body weight	0.21 ± 0.02	0.16 ± 0.01^a^	0.17 ± 0.02^a^	0.17 ± 0.01^a^
RQ/surface area	1.28 ± 0.10	0.99 ± 0.08^a^	1.06 ± 0.16^a^	1.00 ± 0.07^a^
RMR/body weight (kcal/h.kg)	2.46 ± 0.23	2.54 ± 0.34	2.63 ± 0.27	1.06 ± 0.23^abc^
Fat oxidation (mg/min)	1.38 ± 0.28	3.31 ± 0.57^a^	2.85 ± 1.26^a^	3.42 ± 0.42^a^
Carbohydrate oxidation (mg/min)	1.19 ± 0.25	Not detected	Not detected	Not detected
Energy Balance (kcal/day)	62.93 ± 4.27	62.42 ± 4.60	63.11 ± 4.57	76.55 ± 5.05^abc^

Values are given as mean ± SD.

Ob, Obese rats receiving saline; Ob-Olive, obese rats receiving olive oil; Ob-Oleuropein, obese rats receiving oleuropein; Ob-cafeic, obese rats receiving cafeic acid; VO_2_, oxygen consumption; VCO_2_, dioxide carbon production; RQ, respiratory quotient; RMR, resting metabolic rate.

^a^*p *< 0.05 vs. Ob group.

^b ^*p *< 0.05 vs. Ob-Olive group.

^c ^*p *< 0.05 vs. Ob-Oleuropein group.

### Morphometric and biochemical determinations in the heart

The heart weight, heart weight/body weight ratio, total protein, catalase, GSH-Px and LDH were unaffected by the treatments in both dietary conditions (Tables 4 and 5).

**Table 4:** Morphometric and biochemical determinations in the heart of standard fed rats

	Groups			
	
Cardiac determinations	C	C-Olive	C-Oleuropein	C-Cafeic
Heart weight (g)	0.95 ± 0.14	1.03 ± 0.10	1.00 ± 0.11	1.05 ± 0.05
Heart weight/body weight (g/kg)	2.85 ± 0.17	2.88 ± 0.08	2.90 ± 0.05	2.84 ± 0.21
Total protein (g/g tissue)	0.18 ± 0.03	0.17 ± 0.01	0.17 ± 0.02	0.19 ± 0.01
LH (nmol/g tissue)	150.07 ± 9.96	135.03 ± 3.18^a^	129.93 ± 6.66^a^	144.20 ± 14.75
TAS (%)	26.78 ± 2.39	31.40 ± 2.04^a^	33.51 ± 3.39^a^	27.96 ± 6.48
LH/TAS	5.81 ± 1.56	4.40 ± 1.04	3.92 ± 0.55^a^	5.60 ± 2.38
SOD (μmol/g protein)	9.90 ± 0.73	8.77 ± 0.82	9.32 ± 0.97	7.6 ± 0.63^ac^
Catalase (nmol/g protein)	985.0 ± 80.7	967.4 ± 49.7	956.5 ± 101.6	887.0 ± 90.3
GSH-Px (nmol/mg protein	6.65 ± 4.95	6.31 ± 3.14	6.93 ± 1.31	5.58 ± 1.93
LDH (nmol/mg protein)	751.5 ± 116.8	781.6 ± 75.7	712.8 ± 235.3	636.6 ± 108.5

Values are given as mean ± SD.

C, control rats receiving saline; C-Olive, standard-fed rats receiving olive oil; C-Oleuropein, standard-fed rats receiving oleuropein; C-Cafeic, standard-fed rats receiving cafeic acid; LH, lipid hydroperoxide; TAS, total antioxidant substances; SOD, superoxide dismutase; GSH-Px, glutathione peroxidase; LDH, lactate dehydrogenase.

^a^*p *< 0.05 vs. C group.

^b ^*p *< 0.05 vs. C-Olive group.

^c ^*p *< 0.05 vs. C-Oleuropein group.

Myocardial LH was lower, and TAS was higher in C-Olive and C-Oleuropein than in C. SOD was lower in C-Cafeic than in C and C-Oleuropein groups (Table 4). Myocardial LH and TAS remained unchanged in hypercaloric fed animals (Table 5).

**Table 5:** Morphometric and biochemical determinations in the heart of obese rats

	Groups			
	
Cardiac determinations	Ob	Ob-Olive	Ob-Oleuropein	Ob-Cafeic
Heart weight (g)	1.05 ± 0.08	1.01 ± 0.08	1.04 ± 0.08	1.04 ± 0.09
Heart weight/body weight (g/kg)	2.67 ± 0.23	2.62 ± 0.24	2.61 ± 0.08	2.62 ± 0.09
Total protein (g/g tissue)	0.16 ± 0.02	0.17 ± 0.01	0.17 ± 0.01	0.18 ± 0.03
LH (nmol/g tissue)	127.89 ± 7.57	135.00 ± 2.28	127.95 ± 9.32	126.83 ± 8.69
TAS (%)	30.89 ± 3.55	30.12 ± 1.44	30.24 ± 3.54	31.02 ± 3.54
LH/TAS	4.19 ± 0.63	4.49 ± 0.26	4.30 ± 0.75	4.15 ± 0.68
SOD (μmol/g protein)	9.56 ± 0.92	8.95 ± 1.14	9.78 ± 0.31	9.06 ± 1.36
Catalase (nmol/g protein)	973.1 ± 126.1	1084.3 ± 99.5	1039.0 ± 75.1	1064.3 ± 235.3
GSH-Px (nmol/mg protein	5.90 ± 2.44	5.37 ± 1.50	6.55 ± 1.26	5.80 ± 0.54
LDH (nmol/mg protein)	780.0 ± 83.4	878.9 ± 137.8	758.6 ± 96.8	792.2 ± 71.8

Values are given as mean ± SD.

Ob, Obese rats receiving saline; Ob-Olive, obese rats receiving olive oil; Ob-Oleuropein, obese rats receiving oleuropein; Ob-cafeic, obese rats receiving cafeic acid; LH, lipid hydroperoxide; TAS, total antioxidant substances; SOD, superoxide dismutase; GSH-Px, glutathione peroxidase; LDH, lactate dehydrogenase.

^a^*p *< 0.05 vs. Ob group.

^b^*p *< 0.05 vs. Ob-Olive group.

^c ^*p *< 0.05 vs. Ob-Oleuropein group.

**Figure 1:**
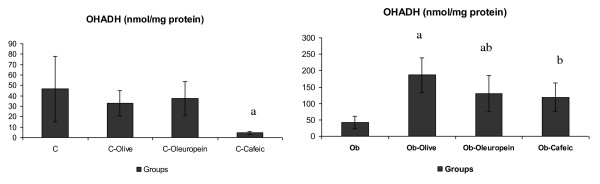
**Beta-hydroxyacyl coenzyme-A dehydrogenase (OHADH) activity in cardiac tissue of rats**. C, control rats receiving saline; C-Olive, standard-fed rats receiving olive oil; C-Oleuropein, standard-fed rats receiving oleuropein; C-Cafeic, standard-fed rats receiving cafeic acid; Ob, Obese rats receiving saline; Ob-Olive, obese rats receiving olive oil; Ob-Oleuropein, obese rats receiving oleuropein; Ob-cafeic, obese rats receiving cafeic acid. Values are given as mean ± SD. ^a ^*p *< 0.05 vs. respective group. ^b ^*p *< 0.05 vs. respective Olive supplemented group. ^c ^*p *< 0.05 vs. respective Oleuropein supplemented group.

**Figure 2:**
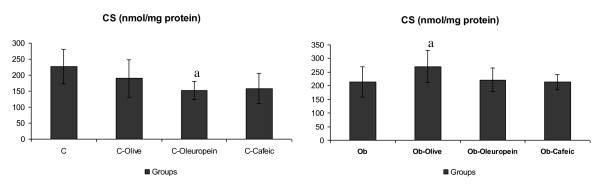
**Citrate synthase (CS) activity in cardiac tissue of rats**. C, control rats receiving saline; C-Olive, standard-fed rats receiving olive oil; C-Oleuropein, standard-fed rats receiving oleuropein; C-Cafeic, standard-fed rats receiving cafeic acid; Ob, Obese rats receiving saline; Ob-Olive, obese rats receiving olive oil; Ob-Oleuropein, obese rats receiving oleuropein; Ob-cafeic, obese rats receiving cafeic acid. Values are given as mean ± SD. ^a ^*p *< 0.05 vs. respective group. ^b ^*p *< 0.05 vs. respective Olive supplemented group. ^c ^*p *< 0.05 vs. respective Oleuropein supplemented group.

C-cafeic rats had the lowest OHADH activity. Ob-Olive, Ob-Oleuropein and Ob-Cafeic rats had higher OHADH than Ob (Figure 1). CS was lower in C-Oleuropein than in C, as well in Ob-Olive than in Ob group (Figure 2).

## Discussion

Dietary olive oil supplementation [20], and more recently olive oil phenols [21] have been recommended as important therapeutic interventions in preventive medicine. However, a question remains to be addressed: what are the effects of olive oil and its minor phenolic compounds on obesity-induced cardiac metabolic changes?

To the best of our knowledge this is the first study that evaluated the relative potency of olive oil and its phenolic constituents, oleuropein and cafeic acid, on some markers of metabolic pathways in cardiac tissue of obese rats, as well their relationships with calorimetric parameters and oxidative stress. The present study brought new insights into the long term and low-dose intake of olive oil and its compounds on cardiac energy metabolism.

To demonstrate the efficiency of obesity induction [19,34] despite the same food intake, Ob rats showed higher final body weight, surface area and BMI than C (Table 1). Prospective studies [35] demonstrated that a relatively low RMR and a high RQ [36] are predictors of body weight gain. In fact, as previously observed [34] obesity was characterized in Ob rats from reduced RMR, oxygen consumption, VO_2_/final body weight and VO_2_/surface area, corroborating with lower fat oxidation (Table 1). There was also higher energy balance indicating that energy intake exceeded energy expenditure for vital functions (RMR), thus providing greater amount of energy to be stored as fat.

The detected fasting carbohydrate oxidation in Ob group was associated with liver glycogen storage due hypercaloric diet intake in these animals. Since the influence of the meal had essentially ended in the post absorptive state, the fasting RQ value gives an indication of the glycogen storage [27].

As previously found [20,21], in both dietary conditions there were no significant alterations in morphometric parameters by olive oil and its phenolic compounds. There were no significant changes in heart weight, heart weight/body weight, as well as in cardiac protein, indicating adequate protein supply in both dietary conditions.

The lower RMR in C-Oleuropein rats, as well the higher RMR in C-Olive and C-Cafeic rats were not enough to significantly change the energy available for fat storage (Table 2). Note that in C-Olive and C-Cafeic rats there was a better fat utilization and this was evidenced by the lower RQ [34] in these animals. The detected carbohydrate oxidation in C-Oleuropein rats was associated with the highest RQ, RQ/body weight and RQ/surface area found in these animals (Table 2). Therefore, the lower fat oxidation in C-Oleuropein animals was not surprising, since the change in fuel selection is controlled by carbohydrate intake, and when carbohydrate oxidation rises in response to intake, there is a profound counterregulatory suppression of fat oxidation, because triacylglycerol lipase enzyme from the adipose tissue is inhibited by the insulin secretion [11]. Oleuropein enhanced glucose uptake in tissues, increasing insulin response [6] and the glycogen storage [36].

It was widely accepted that oxygen consumed for carbon dioxide liberation, during substrate oxidation depends upon the oxygen amount in dietary nutrient. Because fat oxidation requires more oxygen, there was a significant reduction in the RQ, RQ/body weight and RQ/surface area in Ob-Olive, Ob-Oleuropein and Ob-Cafeic rats (Table 3).

Several mechanisms may be associated with increased fat oxidation in obese rats receiving olive oil and its phenolic components. It is well known that absorption and delivery of dietary compounds by intestinal cells are part of a complex process, which is influenced by the physiological state of enterocytes. A persistent situation of redox imbalance, due to hypercaloric diet intake has been associated with gastrointestinal alterations [37]. Under these conditions, it might be expected an imbalance in the absorption of dietary compounds and the metabolic utilization for energy generation [38]. Olive oil and its compounds modulating cellular signal [39] and the activity of peptidases [3,40] would allow the adequate balance between uptake and metabolism of dietary compounds.

Judging from our experimental results, it was evident that the beneficial effects of olive oil and its minor constituents enhancing fat oxidation were reflected in cardiac tissue of obese rats. Note that there was no antioxidant activity of olive oil and its phenolic compounds in cardiac tissue of animals fed with hypercaloric diet.

The enhanced fat oxidation was demonstrated in cardiac tissue by higher OHADH and CS activities in Ob-Olive rats (Figure 1 and 2). OHADH is a key enzyme for fatty acid oxidation, and CS is the key enzyme for the control of the flux of metabolites through tricarboxylic acid cycle. A clear link between triacylglycerol accumulation and the cardiomyopathy was established in experimental models in which the rate of fatty acid uptake by the heart was increased, or the capacity for fatty acid oxidation was reduced in the mitochondria [41]. Myocardial OHADH activity was also significantly enhanced by oleuropein and cafeic acid in obese animals, despite the maintenance of CS activity in these animals. It has been shown that hypercaloric diet induces adverse effects on cardiac function through changes in fatty acid metabolism, by inappropriate activation and expression of PPARα (peroxisome-proliferator-activated receptor α) as a result of glucotoxicity. The decrease in the transcription of PPARα may be a regulatory event for the reduced use of fatty acids [42]. Thus, cafeic acid and oleuropein could allow adequate PPARα activation and fatty acid utilization, increasing the OHADH activity under obesity conditions, reducing triacylglycerol accumulation in cardiac muscle. Optimizing cardiac energy metabolism in obese conditions may be one approach to prevent and treat cardiac dysfunction [11,13].

Curiously, no significant changes were found in myocardial metabolic enzymes of C-Olive rats, whereas, CS was significantly reduced in C-Oleuropein rats, despite no changes in OHADH (Figure 1 and 2). This indicated enhanced glycolytic pathway relative to aerobic metabolism, or delayed flux of metabolites through tricarboxylic acid cycle in cardiac tissue. Glycolysis preferentially serves energy channeling to sarcolemmal membranes, were glucose transport into cells occurs, by providing this readily available substrate for glycolytic enzymes bound to sarcolemmal molecular complexes [13]. Glycolytic pathway thus represents low capacity, but high specificity modules of the integrated metabolic network of a cardiac myocite [11]. On the other hand, the reduced OHADH, and the maintenance of CS activity, clearly indicated that C-Cafeic rats had depressed fatty acid degradation, relative to aerobic metabolism in cardiac tissue, as compared to C, C-Olive and C-Oleuropein rats. Therefore, in standard fed conditions, dietary supplementation with olive oil phenolic compounds induced changes in the substrate used for energy generation in cardiac tissue. Further studies may be considered to clear this open question, and to show the importance of these changes on cardiac function.

Anyhow, the beneficial effect of olive oil in standard-fed condition was evidenced from the reduced myocardial LH in these animals (Table 4). Lipids accumulation within the myocardium induces cardiac lipotoxicity [12,13]. Oxidative stress and lipotoxicity with increased LH may affect myocardial function, in a fashion that mimics reperfusion injury including persistent cellular loss of K^+^, depletion of energy phosphates and decreased metabolic function [43]. Note that despite the effects of cafeic acid reducing myocardial SOD, no significant changes were found in LH and TAS concentrations as compared with C rats.

Although both oleuropein and olive oil have reduced LH, the antioxidant responses in both groups occurred by different ways. The antioxidant defense system includes SOD that catalyzes the destruction of superoxide radical (O_2_^_^) by dismutation and hydrogen peroxide formation, catalase and GSH-peroxidase that catalyzes the conversion of hydrogen peroxide to water. The total antioxidant substances (TAS) include non-enzymatic, lipophilic and aqueous antioxidants [11]. Enhanced TAS was associated with direct antioxidant activity of olive oil and oleuropein, because of the free radical scavenger ability [44]. This property depends on the number of hydroxyl radicals in the phenolic molecule [21]. Oleuropein and hydroxytyrosol, free radical scavengers, have antioxidant activity as found in other antioxidants, such as vitamin E and butylated hydroxytoluene (BHT) [20]. On the other hand, it has been demonstrated that olive oil may interfere in iron absorption [24]. Considering the importance of iron in the oxidative stress induction, the reduced iron uptake and diminished serum concentration of iron may be alternative mechanism for reduced myocardial LH in C-Olive animals.

## Conclusions

In conclusion, the present study brought new insights of olive oil effects on obesity related cardiac metabolic changes, demonstrating for the first time that olive oil enhanced fat oxidation and regulated myocardial metabolic enzymes, optimizing cardiac energy metabolism in obesity conditions. Olive oil and its minor phenolic compounds, oleuropein and cafeic acid had myocardial antioxidant activity in standard-fed conditions.

## Competing interests

The authors declare that they have no competing interests.

## Authors' contributions

**GMXE **took part in planning the study design, sample analysis, analyzed the data and involved in drafting and writing of manuscript. **FRFS **contributed to the study design, sample analysis and performed the statistical analysis. **KKHRR **and **GSA **involved in drafting and sample analysis. **ELBN **took part in planning and supervising the study design, contributed to the analysis, writing of manuscript, edited the paper, revising it critically for important intellectual content and provided the final version. All authors read and approved the final manuscript.
